# A Review of the Role and Challenges of Big Data in Healthcare Informatics and Analytics

**DOI:** 10.1155/2022/5317760

**Published:** 2022-09-29

**Authors:** Banan Jamil Awrahman, Chia Aziz Fatah, Mzhda Yasin Hamaamin

**Affiliations:** Technical College of Applied Science, Sulaimani Polytechnic University, Sulaimani, Kurdistan Region, Iraq

## Abstract

Healthcare has evolved with the development of technology to improve the quality of life and save lives. Today, big data is considered as one of the most essential and promising future technology areas and has been attracting the medical community's attention. As a result of big data, we can improve patient outcomes, personalize care, improve relationships between the patient and the provider, and decrease hospital costs. The effect of big data is very large since medical societies are known for their size, diversity of complexity, and a high degree of dynamism. Big data has been discussed from different viewpoints in recent years, protecting its involvement in many aspects, specifically those related to the healthcare system. Assembling health information, sharing data, and integrating health are essential in spreading health care. In addition, the security and privacy of data are critical since the data must be accessed from multiple locations within the distributed system. This paper review aims to understand the role of big data in healthcare issues aggregating data and the challenges associated with big data in healthcare. The papers that have been selected for review are from last year's research.

## 1. Introduction

Proper medical treatment for specific diseases will improve patient outcomes and decrease life-threatening conditions. It also reduces the side effects of drugs that impact their lives and medical waste products. Finding new drugs and equipment leads to further accuracy in the healthcare system [1]. Some sorts of medical equipment especially those which are continuously wearable record data and the high-velocity data requires fast processing; in a specific data source, the value may be limited, but in the public sector, it may get to a maximized value through fusion of electronic health records (EHRs) and electronic medical records (EMRs) [2]. CT scan for visualizing a patient's body, for instance, a patient's abdomen, is a plentiful source of high measurements data showing the abdomen with tiny details in such a high resolution that it is too beneficial in clinical settings and research for discovering abdominal features [3]. Web/mobile applications in health care have been expanded that enable patients to send their signs and symptoms to the provider; those applications contain fundamental diseases, first aid, types of drugs, and also direct the patient to the specialist [4]. Health care system collects real-time biomedical signals (e.g., ECG, pulse oximetry, and blood pressure) in different places on mobiles, a healthcare application is installed, and health data are synchronized for analysis and storage by a cloud computing system [5] in health care; big data can be represented with the assistance of progressed information technology which observes information to make policy-making better; and a life chart can be used to research medical expenses and population aging, which applies evidence of policy-making [6]. Health care costs will be elevated with the aging population; Japan has begun using big data technologies for approaching and managing elderly persons, and big data analytics is used to attain information from complex and enormous datasets obtained from data mining [7].

This review provides a concise analysis of some productive efforts. In addition to the drawbacks and advantages of these technologies, privacy and security have been discussed in phases of big data analytics in healthcare big data. Big data analytics has bridged the distinction between organized and unstructured data. The transition to an integrated data environment is a recognized hurdle to overcome. Big data's objective and guiding concept is to gather more information and more insights from this information and has the capacity to forecast future occurrences. Several reputable healthcare firms expect a robust growth rate in the healthcare data sector.

## 2. Literature Review of Healthcare Data

**Figure 1:**
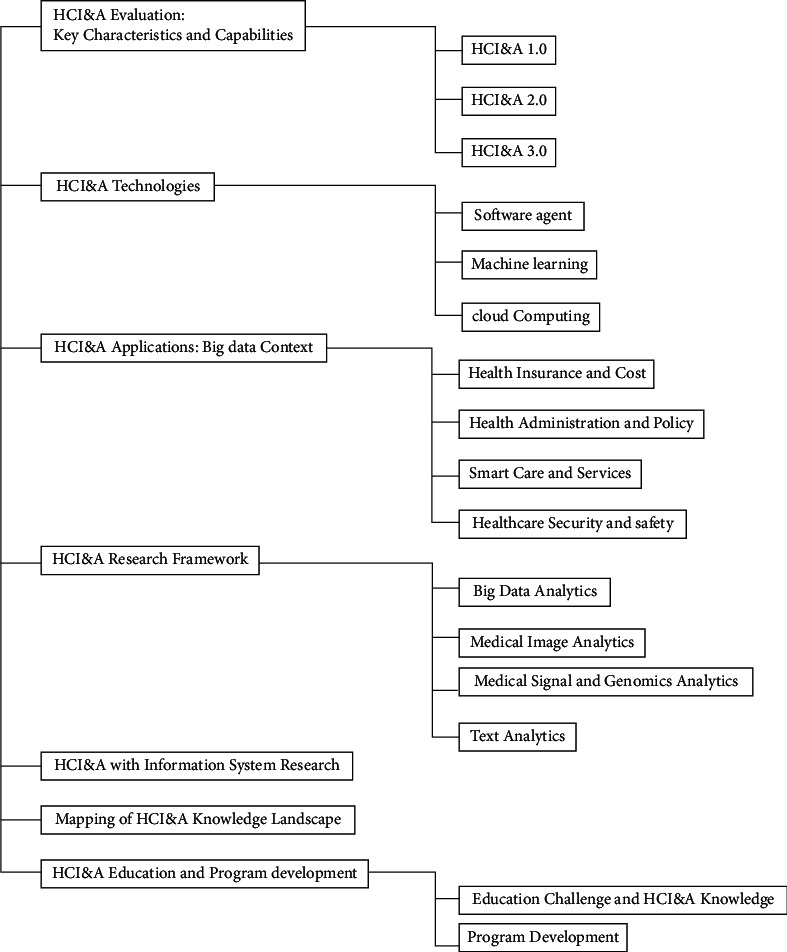
Overview of the healthcare informatics and analytics landscape: standards, technologies, applications, and emerging research.

Multiple forms of healthcare data include biomedical signals, genomic data, sensing data, biomedical images, and social media [8]. Genomic data analysis lets someone realize more about genetic markers, disease condition, consanguinity, and mutations; clinical text mining converts data from practical medical notes from disorganized format to applicable information, extraction of information, and natural language processing which extract helpful information from the massive volume of practical text. Social network analytics such as Web logs, Twitter, Facebook, social networking sites, and search engines helps to discover new health methods and worldwide health issues and trends based on different social media sources [9] before analyzing the severity of the disease; therefore, reasonable diagnostic patterns should be used.  Table 1 represents the diagnostic plan for a definite diagnosis of the disease; the diagnosis is based on three conditions (frequency, pattern, and scale) [10].  Figure 1 identifies the importance of digitized health-related information we create; this figure contains seven layers showing personal health data. The privacy of individuals should be protected in this survey [11].

This is about wearable functions of the physiological sensors, and then referred to as mobile physiological sensor systems, designed for gathering user information through different sensors. These sensors measure a patient's vital signs, including ECG, temperature, oxygen saturation, pulse rate, and blood pressure. After that, those real-time data will be shown on the user's smartphone and sent to the health care cloud. Cloud systems can analyze and make classification using machine learning methods and store information in a private and secured manner. [12]. Healthcare data has been extended with a continuous stream of recent data elements and relationships, various data ranges from individual health information to epigenomes, and copious integration approaches are accepted, such as view integration (emerging and bringing together various databases), link integration (presentation in a web page), warehousing (setting data into a common database), and mash-ups (making a new web application from more than one web-based resource). All these methods make joining data flexible in many ways across sources but nevertheless consist of inadequate computable joined data or integration [13].

## 3. Healthcare Informatics and Analytics (HCI&A) Version 1.0

**Figure 2:**
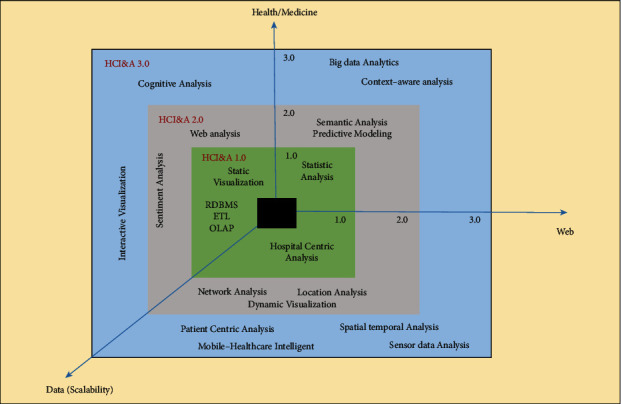
Analyses of health, web, and data in the context of health care.

With the widespread adoption of database methods by different healthcare settings in the 1970s, HCI&A arose from the context of data management and analytics [13]. The goal of the Coral Gables Variety Children's Hospital's Patient Order Management and Communication System (POMCS) during that time was to accomplish three goals: raise income, increase employee productivity, and save money. [13]. These data management systems largely depend on technology for collecting, extracting, and analyzing health data. From a data-centric perspective, HCI&A may be compared to HCI&A 1.0, in which data is wholly organized, homogeneous, and stored in relational database management systems (RDBMS). In addition, three other significant factors contributed to the medical domain's artificial intelligence and data analytics: the medical domain, the web, and data (see Figure 2). It consists primarily of Web 1.0 technologies, Health 1.0 apps, services, tools, and Medicine 1.0 solutions.

A hospital or healthcare institution distributes content on Web 1.0 without interacting with patients; it is primarily an online content repository. In the context of healthcare, Web 1.0 aims to create an online presence for healthcare providers that makes their information available at any time to all clients (primarily patients). In its cover of Web 1.0 tools and methods, HCI&A 1.0 encompasses the fundamentals of web technologies (HTML and HTTP), emerging web technologies (XML), and hypertext. Consumers and service providers cannot be involved in HCI&A 1.0 technologies. Provider-centric approaches are at the center of Medicine 1.0 and Health 1.0. Database technologies such as warehouses are used to integrate healthcare data management systems. Various statistical tools and data mining tools are also available in HCI&A 1.0 to classify, segment, cluster, and analyze health data. The leading commercial healthcare informatics systems from IBM, Oracle, and Microsoft already incorporate some HCI&A features. In addition to extracting, transforming, and loading data, we also have OLAP, database querying, data mining, and visualization packages within HCI&A 1.0. However, the software must also be able to perform some intelligence and analytical tasks.

## 4. Big Data Analytics in Healthcare System

Healthcare data digitization is the result of big data development and revolution. The rapid growth in data over the past few years led to the announcement of a new domain called big data. In information technology, the term “big data” is usually used to express enormous data that are too big and hard to deal with by the traditional database [14]. Intelligent healthcare systems, including big data analytics, make new and mobile health, saving medical costs and expanding efficiency [15]. Predicting pharmaceutical outcomes by predictive analytics, people who get the most benefit from pharmaceutical interventions are recognized, making pharmacists understand more about the side effects and risks of the medications [16]. Handling precision medicine is done by data collecting and management (sharing data, storing data, and privacy) to analytics (data merging, data processing, and visualization); compound and complex biomedical data which are enormous are becoming accessible due to biotechnologies progression, and analytics of big data is acquired to use these different data. It covers application sectors such as imaging, health, sensor, and bioinformatics [17]. For big data analytics, accuracy is essential; personal health records (PHRs) may contain typing errors, abbreviations, and mysterious notes; medical personal data input may contain errors, or it may be put in the wrong environment, which affects the efficacy of the collected data instead of getting uploaded by the professional trainee and medical practitioner in a clinical environment, and gathering data from social media may result in inaccurate prediction [18]. Fast-growing noise data is a significant problem; heterogeneous results are caused by various degrees of quality and completeness, which leads to false discoveries; there are two main problems, which are the inappropriate quality of data and biases because of absent randomization; big data value elevation are made by connecting various and analyzing all existing data [19]. Big data depending systems have progressed, including patient discharging records, electronic certificates of death, and medical claim data, which use the coding of International Classification of Diseases (ICD), and using big data courses in strategies of surveillance from the internet and social media has been preferred [20]. Data technologies like SQL databases have established healthcare processing. Some features like rational relationships and local access between logical and physical data spreading are significant to upgrading and performing parallel processing in database distribution [21].

Clinical and molecular information has been proposed in a big data-driven approach. Therapeutic medications and biomarkers are spotted in the approach. Following preclinical or clinical accuracy is accomplished by cross-species analysis; hence, the cost and time of biomarkers and therapeutics are decreased [22]. The primary function of the warehouse is for structured data and has a set of modules for unstructured data analysis. Initial accomplishments of substructures or frameworks were built for a big data paradigm. The framework used a Hadoop cluster for running modules, and distributed counting ability is used in big data according to research [23]. Utilization of the enormous data storage and reliability of the Hadoop big data by the system makes a considerable reduction in storage and upgrade costs. Mobile applications are widespread, keeping doctors and patient users in touch, decreasing complex medical communications and increasing digitalization. Hadoop is a software framework that uses a master-slave. A group of essential background programs is mandatory to get Hadoop running in a completed cluster softly; it is also spread by a large amount of data and progress by Apache Foundation; it is likely to develop a distributed program is capable of dividing a large amount of program into small working units, making the cluster's ability to make high-speed storage [24]. MapReduce is based on rough set theory RST which is used for reducing attributes and includes these procedures for characteristic acquisition and accomplishes them on the MapReduce parallel large-scale rough set method which is used in runtime systems like the Phoenix, Twister, and Hadoop to get features from the big database by data mining two acceleration of computation of equivalence classes ae done by using the framework structure of the (key, value) pair; MapReduce parallelizes traditional attribute reduction [25]. Tables 2 and 3 represent big data tools for health care [17].

Industry precised medicine is a sort of big data application in health issues, including the manufacturing of medical drugs and devices; it is considered as a strategic plan; this application benefits from IoT, industry, and multitopic. It has been suggested that it makes sense of big data with artificial intelligence, next-generation technology, and IoT [26]. Based on IoT technology, an intelligent healthcare framework has progressed for anyone during workouts; the Bayesian belief network uses an artificial neural network model to predict a patient's health-related susceptibility. There are four critical areas of big data analytics: model development, business models, data management, and visualization [10].

## 5. Challenges of Big Data in Healthcare Systems

Big data has been evolving, introducing challenges and problems caused by the exponential growth of healthcare data. The constant changes of big data present many challenges in analyzing, storing, and recovering huge amounts of data. Conventional or standard database systems cannot be used to process, store, and take information due to their massive and enormous volume [27]. Big data issues that generally happen in healthcare organizations are covered by four main categories [28, 29]: a huge amount of unstructured data are included in big clinical data like handwritten data and natural language, a reasonable degree of difficulty is brought by clinical big data's analysis, integration, and storage. It is insufficient for agencies to share structured data, and unstructured data sharing among organizations is more complicated. It is a great challenge how to effectively mine an enormous amount of unstructured data. Big data has some characteristics. One of them is variability in data sources; medical data has potent timeliness; having appropriate moments of medical care is an example.

In the medical industry, data processing speed is in great demand particularly while patients' situation deteriorates quickly. The data privacy and security of the patients and ill persons are influenced by challenges and disputes with these real-time applications like cloud computing used to analyze data. Recently cloud computing has offered new possibilities for medical big data mining and sharing. Before cloud computing can become even more practical, several challenges must be overcome. [30]. First, cloud computing offers a simple and flexible way to mine resources. However, it elevates the risk of privacy disclosure. It is a fact that is clinically evident in clinical informatics. Second, importing or exporting an enormous amount of data in medicine to the cloud (petabyte). Network bandwidth increases the cost of data and restricts the speed [31] (see Table 4).

### 5.1. Economic Challenges

The medical field facilities of patients and health care providers such as doctors are dependent on paid services. It disproportionately negatively impacts technology advancements in connection with this process [32]. *Big Data Technology Challenges.* Being highly fragmented and enormous, big data in health care leads to information quality and technology problems, making it a barrier to accomplishing healthcare vision [33]. Security and privacy issues along with the history of big data include the privacy of healthcare data which is serious because of potentially essential and sensitive information about individual healthcare providers. In order to make healthcare data unavailable in public, it must be secured from unauthorized access, preventing the data from attackers. This means security is the most important task, which is also a challenge [34].

### 5.2. Privacy

The most vital fault is the lack of intimacy and privacy. Big data must have access to almost everything, even social media life and private recordings, to have enough effects. However, because of revealing private information, the price is paid. Moreover, there is no patient freedom. However, there are regulations for stating medical recordings' privacy. However, they are not considered since it is believed that the information of someone should not be forbidden. At the same time, it is related to their health. The privacy risks associated with big data in health care have been stopped in articles such as big data privacy and security in health care [35].

### 5.3. Health Information Systems on the Cloud

**Figure 3:**
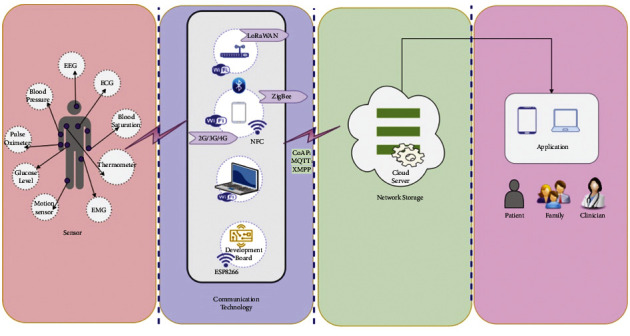
The communication technology utilized by the human body to transmit signals to the cloud.

The adoption of cloud-based platforms has improved and streamlined the design, development, and deployment of clinical information systems, hospital administrative information, and medical images [15]. Several such structures are in place to facilitate data collection (for example, the entities are often provided with mobile user interfaces to cloud services to gather and manage healthcare information). In addition, these systems facilitate information exchange between various medical structures, hospitals, and patients since they integrate data in several ways. The performance of the system is rarely considered. Security and privacy, considered essential, are often at the center of their design (see Figure 3).

### 5.4. Telepathology, Telehealth, and Disease Surveillance

Telepathology services were envisioned as a possible outcome of combining robotic microscopy, video imaging, databases, and the then-new availability of broadband telecommunications in the 1980s. Many contributions demonstrating ICT applications have been presented, illustrating how ICTs can assist with telemedicine, telepathology, and disease monitoring. Research has been conducted on two problems: (1) general frameworks for most cases and (2) studies that focus on particular diseases, such as cancer detection, cardiovascular disorders, diabetes, Parkinson's disease, and Alzheimer's disease. These monitoring systems may then be utilized as a tool for large-scale research and as a means for customizing therapies (as in P4 Medicine). Likewise, surgery is expected to become more transparent in the future. Open surgery operating rooms often use video cameras for lighting. It allows an infinite number of viewers to view the surgical operation. Teleconsultation is possible with these instruments, eliminating the need for the consultant to be physically present. A remote consultant may use telepresence during surgery if an active camera holder is used and the remote consultant can move the camera. It is physically impossible for the surgeon to see the operating room when telesurgery is used. The availability of limited virtual pathways to fog services at the edge could assist in closing the gap when best-effort Internet connections are insufficient for some types of applications (e.g., to recreate the effect of a microscope locally). Providing remote federated sites with tools for offloading sophisticated image processing and data mining operations, it may, for instance, allow remote federated sites to cooperate on nontrivial diagnoses without experiencing increased cloud access latency (see Table 5).

## 6. Big Data Management in the Healthcare System

**Figure 4:**
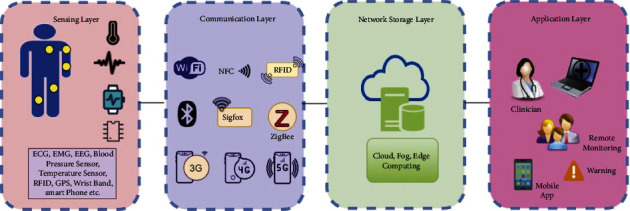
A four-layer system for IoT-based health monitoring.

Healthcare activities generate large amounts of data. Analytical procedures should be used to derive actionable judgments from data management technologies. This section is divided into five subsections: machine learning-based, agent-based, cloud-based, heuristic-based, and hybrid-based. Further, in this section, the chosen articles are described in their approach, differences, advantages, and drawbacks (see Figure 4).

This section examines the most common machine learning techniques for managing extensive healthcare data along with their fundamental characteristics. In the last few years, machine learning methods have been used to process large amounts of data based on artificial intelligence methods and historical databases. Therefore, machine learning techniques can be compellingly applied to this problem [31].Machine learning algorithms play a significant role in managing massive biomedical data based on current issues in biomedical data [32].

## 7. Discussion on Intelligent Health Care

Sensor data are primarily unstructured in intelligent health care. Sensor-based health and wellness monitoring generate unstructured data beyond the human ability to process and analyze manually. There is a huge gap between the potential and the utility of such an enormous amount of unstructured data. The vast amount of unstructured data from streaming sensors is useless due to its variability and complexity. Data analytics pipelines for intelligent healthcare applications follow a similar process to the standard analytical method. Data management, processing, and finding are critically important in health care [33, 34]. The correct data must be collected at the right time and context for an effective data discovery process. There needs to be an end to the division between numerous fields, such as medical science and computer science, for context-awareness in healthcare applications [35].Data curation is, therefore, more useful when addressing effective data discovery when it comes to improving knowledge of patient physiological and psychological care.

### 7.1. Interpretation of Data

Predictive analytics may be more effective when combined with structured and unstructured EHR data. Clinical events can be extracted from EHR data, and comparable phrases can be categorized in semantic space. By concatenating their representation using semantic space, structured and unstructured data are integrated more efficiently than if the occurrences are represented separately. Using semantic spaces to extract clinical language from EHR, diverse and distributed representations can predict clinical outcomes effectively. The lack of an agreed-upon standard for terms, acronyms, and abbreviations further complicates the semantic categorization of datasets. This factor may impair the effectiveness of semantic classification based on similar terms. Various types of information can be collected from health records for purposes such as pharmacovigilance, phenotyping, and illness detection. Data from EHRs, EMRs, PHRs, and omics provide a wealth of information for many different medical fields. However, they should also be used to enhance healthcare. The model has been evaluated through interviews with domain experts, following the combination of clinical and genomic data for deep cancer phenotyping. In this study, real-time datasets could neither be used to assess the representation standard nor assess the suggested model. A robust knowledge base and accurate data modeling may facilitate using unstructured clinical notes from multiple institutions. The interpretation of data is equally important as obtaining usable information from various forms of health records, and this is called enhanced unstructured data analytics.

### 7.2. Quality of Data

The literature has identified that several quality parameters can be used to enhance and assess significant data quality, such as correctness, completeness, consistency, timeliness, objectivity, interpretability, and accessibility. Unstructured, heterogeneous, and noisy data add to the difficulty of this task because of their heterogeneity, lack of structure, noise, and the lack of a preset model. In addition to understanding psychological disorders, social media analytics helps to understand society's most prevalent illnesses. Social media analytics has most of the quality issues compared to other fields because postings, reviews, and comments cannot be standardized. Several linguistic issues impede clean analytics. It may be possible to increase analytical efficiency by using hashtags. However, computer, media, and healthcare knowledge are necessary to understand healthcare social media better. As part of effective healthcare analytics, database aggregation and data cleansing may reduce data heterogeneity, lack of structure, and other quality challenges.

## 8. Conclusion

This paper is a brief discussion of some successful work. Privacy and security have also been presented in phases of big data analytics along with the faults and benefits of these technologies in big healthcare data. Big data analytics has held the gap between structured and unstructured data. A well-known obstacle to overcome is the shift to an integrated data environment. The aim and principle of big data are gaining more information; more insights from this information and the ability to predict future data healthcare market show a rapid growth rate which several reliable healthcare companies project. However, in a short time, we have seen a range of analytics in use which has shown improvement effects on health care industry decisions. Computational experts have been forced by the exponential growth of medical data from different domains to design strategies to interpret and analyze various amounts of data. In every area, big data challenges are as follows: storing, searching, capturing, sharing, and analyzing data. Some extra challenges include real-time processing, data quality, privacy and security, and heterogeneous data. Also, healthcare data standards are among the challenges of big data analytics in healthcare systems. [36].

## Figures and Tables

**Table 1:** The scheme for diagnosis of diseases in a system.

Disease and illnesses	Diagnostic method bases	Health measurements
Diabetes mellitus	Scale-based, frequency-based	Blood glucose
Pulmonary disease	Scale-based, frequency-based	Oxygen saturation
Cardiac disease	Pattern matching, frequency	ECG
Infectious disease	Frequency-based, scale-based	Temperature
Hypotensive disease	Frequency-based, scale-based	Arterial pressure
Gastrointestinal tumors	Frequency-based	Video capsule pill

**Table 2:** Represent a list of several large companies which provide and supply services on big data analysis in the healthcare sector [1].

Data	Software	Description
Data integration or dataset or data source	Kafka, Sqoop	Tiny biosensors are placed on patients' body for collecting vital signs data in health applications, and the vital signs include blood pressure (BP), systolic and diastolic, respiratory rate (RR), heart rate (HR), oxygen saturation (spo2), and body temperature (BT)
Data decision and data storage	Apache Spark, Hadoop HDFS	That is responsible for storing data and processing it. That layer consists of two main tools, i.e., Hadoop and Apache Spark, also processing two data algorithms, patient archiving, emergency management, and clinical responses
Emergency detection and clinical response algorithm	Early warning score (EWS)	To verify abnormal situations
Patient classification and disease diagnosis	Machine learning algorithm	Machine learning tool and advanced analytics of huge datasets at high speed. Big data workspace tools are stored on Hadoop clusters for pattern insights discovered from massive data. Solutions to big data use cases by predictive analytics through platforms
Data retrieval and visualization	Hive, Spark SQL	Medical staff can access patients' records using the last platform for storing their data in HDFS and Hadoop. It comprises two data retrieval (spark SQL and hive) and just one graphing tool (Matplotlib). Obtaining data from the Hadoop storage system, which uses a set of criteria defined via queries, is based on the data retrieval tools. The retrieved data is usually stored in a file or displayed on a screen. Using graphics or plots statistically for data visualization is a graphical representation of the retrieved data; in our platform, each tool has been highlighted

**Table 3:** A scheme for some companies using big data.

Company name	Company place	Description	Company sits
Flatiron Health	New York, New York	Enough amounts of data points are utilized by Flatiron Health from cancer patients to develop research and obtain new patient care. This solution enhances all users, such as an oncologist, academic people, and researchers of life sciences connected to the treatment of cancer patients, as well as enables more learning from them	https://www.flatiron.com
Tempus	Chicago, Illinois	The most extensive library for clinical and molecular data in the world is Tempus which aims to provide more clinical context by medical professionals for cancer patients, and this platform is for collecting and organizing data from many aspects of oncology like pathology images, lab results, clinical notes, radiology scans, and oncology research acceleration and also assisting doctor specialists which helps them to make more informed treatment plans	https://builtinchicago.org
Pieces Technologies	Dallas, Texas	Collecting data from everything related to patients to make improvements in cost of care and quality is done through Pieces Technologies which is a cloud-based software company	
PeraHealth	Charlotte, North Carolina	For a patient's overall health, there is a universal scoring system that is a creator of the Rothman index; a peer-reviewed score collects the data in the electronic healthcare system, lab results, vital signs, and nurse assessment. A visual graph provides the score in real-time to recognize any changes and details also to avoid any complications for the patient	
Amitech	St. Louis, Missouri	To apply health data from modern data management to healthcare analytics, Amitech is used. It is specifically used to gather data for people health management solutions and collects physical health data in combination with behavioral health data to recognize risks and let the patients know their health	
SCIO Health	Hartford, Connecticut	For improving patient health, SCIO Health is used, which uses proprietary algorithms and integrated data for providing insights and solutions. The technology detects gaps in care that worsen health outcomes and cause more costs. Identification of these gaps assists medical professionals in detecting risky group patients and avoiding complications and insignificant hospitalization	
Hortonworks	Santa clara, California	Hortonworks are used for pharmaceutical data by pharmaceutical companies and researchers to obtain a better view. Companies can answer questions that were not possible previously because of billions of integrated records. This sparks much more effective research for clinical trials, faster marketing, better yields, and improved safety	

**Table 4:** HCI&A used in various healthcare installations.

Empty cell	Health insurance and cost	Health administration and policy	Smart care and services	Healthcare security and safety
Data	Transactional documents payment album financial statement of the provider, user-generated content, and medical claims data	Official norms and regulations, information sources responses, and remarks from various organizations (i.e., doctors, nurses, patients, and other employees)	Computerized medical records (EHR), medical records patient comments and feedback, and molecular data DNA traces medical records (i.e., blood pressure, X-ray, and ECG)	Fraudulent records of data deviations, monetary data, geographic data, and social media records
Analytics	Detection of rare events, emotional evaluation, internet social network, study of statistical information integrating, segmenting, and clustering	Informational integration, administrative data, and ontological analytics textual examination performance, and appraisal rule of categorization and linkage	Mining associations and clusters, ontologies of health social media network, research data amalgamation monitoring and analysis of health, and network evaluation text analysis visualization	Linguistic text analytics, financial information analytics, GPS data evaluation sentiment analytics and social media network analytics, anomalous observation, criminal network investigation, and visualization
Applications	Funding and donation methodologies, recommendation methods, and system of transparent dispersion	Design of a resource, management policy, engagement and involvement of patients	Healthcare administration, support for healthcare decisions, healthcare service assessment, knowledge acquisition, patient vigilance, and universal healthcare	Criminal investigation, healthcare protection, patient safety, and intelligent care, recommendation system, and security administration
Contributions	To improve customer satisfaction, increase transparency and healthcare funding, and ensure responsibility	Enhanced administrative mobility, ensure appropriate actions at the proper time and location, remove congestion, and advocate for a strategy that is effective and efficient	Enhance healthcare (diagnosis, treatment, and therapy), patient-centered health care, develop an uninterrupted system for health monitoring	Enhance health care protection and reliability

**Table 5:** Health conditions and their corresponding sensors and symptoms.

Diseases	Symptoms	Sensors
Stroke	In addition to losing your equilibrium, experiencing facial weakness, feeling numb on one side of your body, having difficulty speaking and interpreting, experiencing blurred vision in one or both of your eyes, and experiencing vertigo (vertigo caused by a severe headache)	Heart rate sensor, EEG, ECG, EMG, EOG, acceleration sensor, Samsung EDSAP, pulse oximeter, respiratory rate, blood pressure, and pulse oximeter
Lung cancer	The symptoms of chronic coughing include bloody coughing, bone pain, breathing problems, chest discomfort, headache, and weight loss	Sensors for measuring pressure, temperature, and acceleration, as well as FET-based biosensors
Blood cancer	Itchy skin, enlarged, painless lymph nodes in body parts, aching bones, fever, coughing, bone pain, and fever are symptoms of lymphoma	Electrochemical biosensors, optical biosensors, CMOS, thermometers, PPG, accelerometers, heart rate sensors, and blood flow sensors
Cardiac	Symptoms include chest discomfort, shortness of breath, weakness, and pain throughout the body	It has a heart rate monitor, pulse oximeter, accelerometer, glucose biosensor, blood pressure monitor, camera (image), microphone, acceleration sensor, PPG, pressure sensor, piezoelectric sensor, electrochemical sensor, and FET-based sensor.
Parkinson's	Symptoms of essential tremor include tremor, stiffness of muscles, slow movements, speech difficulties, mechanical movement difficulties, and difficulty writing	An accelerometer, a magnetometer, a gyroscope, an EMG, an EEG, and a bend sensor

## Data Availability

It is a review paper and does not have any dataset.
